# Hierarchically embedded interaction networks represent a missing link in the study of behavioral and community ecology

**DOI:** 10.1093/beheco/arz168

**Published:** 2019-10-11

**Authors:** P O Montiglio, K M Gotanda, C F Kratochwil, K L Laskowski, D R Farine

**Affiliations:** 1 Département des Sciences Biologiques, Université du Québec à Montréal, Succursale Centre-ville, Montréal, Québec, Canada; 2 Department of Zoology, University of Cambridge, Cambridge, UK; 3 Chair in Zoology and Evolutionary Biology, Department of Biology, University of Konstanz, Konstanz, Germany; 4 Zukunftskolleg, University of Konstanz, Konstanz, Konstanz, Germany; 5 Department of Biology, & Ecology of Fishes, Leibniz-Institute of Freshwater Ecology & Inland Fisheries, Berlin, Germany; 6 Department of Evolution and Ecology, University of California, Davis, Davis, CA, USA; 7 Department of Collective Behavior, Max Planck Institute of Animal Behavior, Universitätsstraße 10, Konstanz, Germany; 8 Department of Biology, University of Konstanz, Konstanz, Germany; 9 Centre for the Advanced Study of Collective Behaviour, University of Konstanz, Konstanz, Germany; 10 Edward Grey Institute of Ornithology, Department of Zoology, University of Oxford, Oxford, UK

**Keywords:** biological interactions, eco-evolutionary processes, gene–phenotype interactions, multilayer networks, nested networks

## Abstract

Because genes and phenotypes are embedded within individuals, and individuals within populations, interactions within one level of biological organization are inherently linked to interactors at others. Here, we expand the network paradigm to consider that nodes can be embedded within other nodes, and connections (edges) between nodes at one level of organization form “bridges” for connections between nodes embedded within them. Such hierarchically embedded networks highlight two central properties of biological systems: 1) processes occurring across multiple levels of organization shape connections among biological units at any given level of organization and 2) ecological effects occurring at a given level of organization can propagate up or down to additional levels. Explicitly considering the embedded structure of evolutionary and ecological networks can capture otherwise hidden feedbacks and generate new insights into key biological phenomena, ultimately promoting a broader understanding of interactions in evolutionary theory.

## Teaser

Interactions are ubiquitous across biological systems. Modeling their consequences requires capturing how units are organized across biological scales: gene and protein interactions shape phenotypic traits within individuals, individuals are embedded within populations, populations within communities, and communities within ecosystems. Doing so reveals how indirect connections among units arise from the structure of connections at higher or lower levels of organization, and how effects at one level of the network propagate across neighboring levels.

## INTRODUCTION

Within a level of biological organization, units (e.g., genes, cells, phenotypic traits, individuals, populations) affect each other’s state and activity via various forms of interaction (e.g., formation of protein complexes, pleiotropy, competition, mutualism), which we will refer to as connections between units. The overall patterns of connections among units shape gene circuits and phenotypic traits, as well as group-, population-, and community-level processes (Hendry 2017; Garcia-Callejas et al. 2018). Understanding the forms and consequences of such connections is a central focus of research disciplines spanning genetics (Civelek and Lusis 2014; Kratochwil and Meyer 2014), development (Davidson and Erwin 2006), behavior (Wilson et al. 2014), ecology (Poisot et al. 2015, 2016), and evolution (Moore et al. 1997; Bijma et al. 2007).

Recent studies have used various aspects of network theory to emphasize the key features of biological connections and their functional consequences. For example, representation of biological systems as multilayer networks can highlight how units are connected through multiple direct or indirect connections that form different interlinked social networks (Ferrera et al. 2009; Kivelä et al. 2014). These layers can include different social relationships among conspecifics as well as relationships between individuals of different species (Silk et al. 2018; Finn et al. 2019), or different types of ecological interactions within an ecosystem (Pilosof et al. 2017). Research on food webs and eco-evolutionary dynamics have highlighted how biological links among populations within communities, or among individuals within populations, can impact ecosystem dynamics (Hendry 2017). Research in genetics has also highlighted how links among genotypic and phenotypic networks can affect evolutionary change (Stadler and Stephens 2003). Here, we emphasize that these concepts have much broader applicability: 1) networks not only span within but also across multiple levels of biological organization, creating direct and less intuitively—indirect connections between genes, phenotypic traits, organisms, etc., and 2) the fact that units at one level of organization are embedded within units at a higher level results in propagation of network dynamics from one organization level to neighboring levels and beyond.

**Figure 1:**
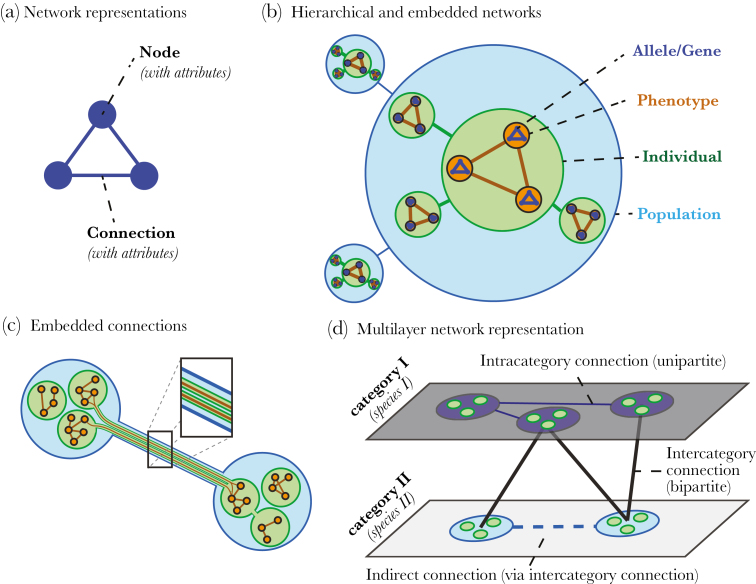
From genes to communities: a framework for describing hierarchically embedded networks of connections. (A) Several biological phenomena can be represented as networks composed of groups of units, or nodes (e.g., genes, phenotypic traits, individuals, populations, communities), and connections between nodes, represented as edges. (B) Networks at one level of organization are embedded into nodes at higher levels of organization. (C) Connections at lower levels are embedded within higher-level connections. That is, lower level connection either determine or are the result of higher-level connections. (D) Within each level of biological organization, multiple categories of nodes and types of connections can exist (e.g., a community contains units representing individuals from two distinct species). These are described as multilayer networks. Interactions among units of different categories (between layers) can create indirect links among units within a given category (within a given layer, dotted line).

Within a hierarchically embedded network perspective, connections among units at a given level of biological organization (Figure 1A) are shaped by—or form the basis for—connections among units at higher and lower levels of organization (Levin 1992) (Figure 1B). For example, trophic or competitive interactions among individual organisms can create connections between their phenotypic traits and genetic network structures (Foster et al. 2017) (Figure 1C). Similarly, emergent collective behavior at organismal scales, shaped by the networks of connections among individuals (Rosenthal et al. 2015), fundamentally shape the patterns of connection at higher levels, such as the dynamics of food webs. As others have explored (Szathmáry and Smith 1995; Melián et al. 2018), hierarchical structure is a fundamental property of biological systems, and it is implicit on our understanding of how, for example, connections at the genetic, molecular, and cellular level within a given organism lead to the emergence of phenotypic traits (Barrett et al. 2008), to how connections among organisms of different species structure ecological communities (Schmitz et al. 2008) (Figure 1D). Analyzing how hierarchically embedded networks operate can clarify short-term mechanistic questions—for instance, how gene–gene interactions can propagate through individual–individual engagements and alter large-scale ecosystem function as well as long-term evolutionary questions such as the adaptive significance of a given trait or behavior. Analyzing how these network structures change through time naturally leads to equally important questions about evolutionary dynamics on longer time scales.

Hierarchically embedded networks have long been proposed in computer science (Engels and Schürr 1995) but are only just becoming more widely considered (Paruchuri et al. 2018). These differ from the related term “nested networks” (Fortuna et al. 2010; Cantor et al. 2017) that instead refers to the idea that nodes can be members of, or “nested” within, a network cluster (Newman 2004; Jordán et al. 2010; Shizuka and Farine 2016). The key difference is that nestedness is defined by the emergent clustering of nodes in a network (e.g., through community detection), whereas hierarchically embedded networks capture the natural organization of units within and across levels of the biological hierarchy. Here, the embeddedness of nodes within other nodes is generally deterministic: the genetic architecture that produces an individual’s phenotype is necessarily embedded within the individual. Thus, our approach is conceptually and structurally different from the common usage of nested networks. Further, hierarchically embedded networks extend the concept of multilayered networks, in that multilayered networks can be embedded within nodes that represent higher levels of social organization.

Here, we discuss the utility of representing hierarchically embedded biological structures. This approach is intended to recapitulate the natural hierarchy of levels of biological organization (Szathmáry and Smith 1995) from both mechanistic and evolutionary perspectives. We outline how hierarchically embedded networks are already present, either implicitly or explicitly, in research across subdisciplines of biology, from microbiology to animal behavior, and from molecular genetics to ecosystem research. However, we suggest that the hierarchically embedded network concept can be applied more formally, and we highlight two key important phenomena that it can capture. First, two or more biological units are often cryptically connected due to mutual interaction partners within or across levels of organization. Second, the innate hierarchically embeddedness of connections in biological networks inevitably causes effect propagation up or down through several levels of organization. We emphasize that this perspective can point to new insights into biological phenomena and encourage research transcending traditional disciplinary boundaries.

## HIERARCHICALLY EMBEDDED NETWORKS CAPTURE BIOLOGICAL ORGANIZATION ACROSS SCALES

Recent work in several disciplines within ecology and evolution has highlighted the value of network representations to understand the outcomes of biological relationships within and across layers of organization in a given system of study. In a generic network representation, biological entities are depicted using groups of units, or nodes (e.g., genes, phenotypic traits, individuals, populations, communities), and connections between nodes, represented as edges (Figure 1A, Table 1), to capture some biological relationship between units. Both nodes and edges can be described by a range of attributes (e.g., sex and age in the case of organismal units, and intensity or frequency in the case of predator–prey interactions). Nodes connected in a network typically represent units at the same level of biological organization, and network connections can be layered to represent different relationships among units or relationships among multiple types of units (Finn et al. 2019). Further, the spatial and temporal dynamics of connections can be mapped in time-varying networks (Rosenthal et al. 2015). In molecular systems biology, network-based approaches have a rich history, for example for gene expression analysis, and metabolomics. Knowledge about gene regulatory networks and metabolic networks has provided insights on genotype–phenotype relationships and how they evolve in response to selection (Stern and Orgogozo 2009; Olson-Manning et al. 2012). The combination of high-throughput sequencing techniques and network modeling has led to major improvements in prediction of microbial species interactions within microbiomes (Faust and Raes 2012). In behavioral ecology, social network approaches have helped quantify the likelihood and speed of transmission of social information (Aplin et al. 2015), or characterize the structure of dominance hierarchies with greater resolution and flexibility (Shizuka and McDonald 2012). At broader scales, studies of eco-evolutionary dynamics consider units spanning from genes to populations and the interconnections among them (Hendry 2017).

**Table 1:** Types of connections within hierarchically embedded networks

Level of biological organization	Example unit categories	Example connections
Subcellular	Genes	The expression of one gene produces a transcription factor that alters the expression of other genes.
	Protein complexes	Many proteins assemble into multi-component structures, such as the flagellar basal body
Phenotype	Behaviors	The expression of parental care behavior is connected to the expression of aggression due to the levels of particular hormones.
	Morphology	The length and shape of one limb is connected to the length and shape of the other limb through genetic and/or physiological mechanisms.
Individual	Bacterium	One bacterium secretes a substance that has a detrimental (or beneficial) effect on another.
	Individual animals	Two female zebra finches are connected by both having mated with the same male zebra finch
Population	Populations of the same species	Two physically isolated populations of crabs are connected to each other via predation by the same population of gulls
	Populations of different species	A population of bacteria is connected to a second population of bacteria because it produces a substance that augments the growth of the second population.
Ecosystems	Community	A terrestrial community is connected to an aquatic community via nutrient cycling processes.

Connections between units can represent a range of direct and indirect relationships. Often, these connections represent the outcome of direct physical contact between two units, such as conjugation between two bacteria. Alternatively, two units can be indirectly connected, for example, if they are influenced by separate interactions with the same third party (Figure 1). Finally, these connections can be temporary (e.g., a mating event between two animals) or more stable (e.g., when individual peptides generate a stable polymer). Here, we list a few examples of potential direct and indirect connections between units at different broad levels of biological organization.

Importantly, networks at one level of organization are inherently embedded into nodes at higher levels of organization (Figure 1B). For example, genotypes—and the protein networks they encode—underlie phenotypic traits, which altogether constitute individual organisms that themselves live and interact together in populations, whose relationship to other populations defines ecological communities. This inherent nestedness means that connections at lower levels can facilitate or contribute to connections at higher levels of organization (see Figure 1C). To take a familiar example, the dispersal of individuals creates connections between populations in the form of gene flow. Connections at higher levels of organization, on the other hand, inevitably lead to connections among their units at lower levels of organization. In a further example, social interactions between two individuals could lead to indirect connections between each individual’s network of physiological states, structural phenotypic traits, and internal networks of gene transcription and translation. The concept we advance here adds to recent insights into how deconstructing multilayered relationships in networks can reveal hidden geometries (Kivelä et al. 2014; Pilosof et al. 2017; Finn et al. 2019) by emphasizing that we can decompose connection patterns at a given level of organization as the result of connections and node properties of units at lower and higher levels of organization.

The details and nomenclature of a network representation will depend on the study system in question, but in general, connections between any two network nodes can be physical and direct (e.g., a mating event between two individuals) or more abstracted and indirect (e.g., exploitation competition between two social groups). Within each level of biological organization, multiple categories of nodes and types of connections can exist (Pilosof et al. 2017; Finn et al. 2019). For example, a community unit can contain units representing individuals of a prey species (Category I) and individuals of a predator species (Category II). Units such as individual organisms are typically connected to others within categories. The advanced insights gained from hierarchically embedded networks is that by capturing connections among different categories (or different types of connections within categories), we can potentially reveal unexpected connections among populations that ordinarily would not be considered to interact with each other. We provide greater mechanistic clarity when identifying counter-intuitive links between individuals, populations, or communities that at first glance appear unconnected by revealing that these can be borne from processes operating at different levels of biological organization. This clarity is arguably the central benefit of a hierarchically embedded network perspective. For example, predator populations that forage across spatially or temporally disconnected prey populations in a food web can generate an indirect link between the prey populations (Bjørnstad et al. 1999). These inter-population indirect connections then allow for the behaviors, structural phenotypic traits, and underlying genetic/development networks of individuals across the prey populations to influence one another on ecological and evolutionary time scales even if the individuals in these populations never interact (per Figure 1A). Such a representation highlights that changes in the behavior of one prey population can propagate across levels by affecting the local predation pressure and, therefore, the pattern of natural selection that the other prey population experiences.

**Figure 2:**
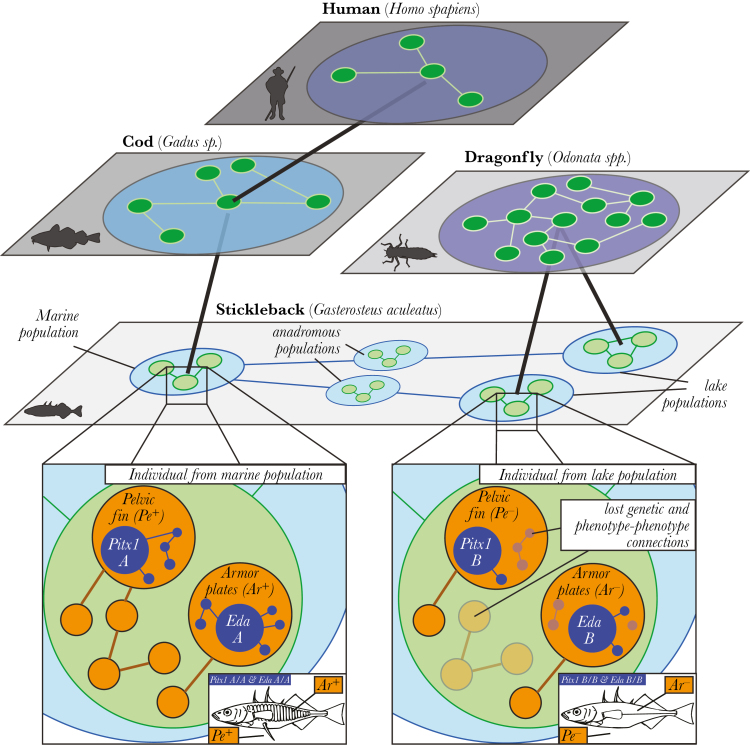
The stickleback system as a case study for hierarchically embedded networks and interactions across hierarchical and species levels. Each plane represents a type of network, with edges linking different nodes that could be genes, individuals, or populations. The different networks are embedded within each other. For example, the genes coding for morphological traits in individual sticklebacks (the *Eda* gene controlling armor plates [Ar], and the *Pitx1* gene controlling pelvic fin [Pe] presence) are embedded within the network of populations of individual sticklebacks which is embedded within a community network, and together, represent a multilayer network. The embedded network structure emphasizes cryptic connections within and among different organizational levels. It therefore facilitates insights into more complex ecological phenomena. Changes in gene frequency of the Eda or Pitx1 locus within one lake could therefore indirectly affect selective pressures in another lake without a direct connection, but through a cryptic, multilayer connection via a predator (here dragonfly).

The interaction structure of a population is determined both by the phenotypic traits of its individual members and the population’s position within the broader ecological community. Thus, many phenotypic traits expressed within populations have cascading effects on community dynamics (Bolnick et al. 2011). For example, Trinidadian guppies from populations that coexist with or without dangerous predators differ in their growth, fecundity, and resistance against parasitism (Magurran 2005; Stephenson et al. 2015). Connections between communities create connections between populations, and eventually individuals, their phenotypic traits, and their genes. Model systems in evolutionary biology, such as the three-spine stickleback, have been used to investigate a large variety of genetic, behavioral, population-focused, and community-level phenomena that can be integrated through a hierarchically embedded network perspective (Figure 2). Another common example of linkages across levels of organization is the dependence of disease transmission on the network structure of hosts, where the connections among hosts depend on the genes (Radersma et al. 2017), phenotypic traits (Mason 2016), and interindividual relationships (VanderWaal et al. 2014), and the transmission dynamic plays out at the scale of populations and communities (Penczykowski et al. 2016).

## CHANGING NETWORKS ACROSS GENERATIONS

Accounting for the natural embeddedness of biological networks, and how and why they can change in time, also promises to deepen our understanding of evolution in natural environments. Within each level of an embedded biological network, changes in the environment can alter 1) the attributes of each unit, 2) the attributes of connections, 3) the pattern (or strength) of connections among units, and/or 4) the number of units. For example, environmental shifts can couple or decouple phenotypic traits, such as growth and survival (van Noordwijk and de Jong 1986); alter the size of groups that the environment can support (Lima et al. 2002); alter the kinds of interactions that occur among populations (e.g., a shift from no interaction to predator–prey interaction when a predator’s other food sources become scarce (Lima et al. 2002); or fundamentally shape meta-community structure (Leibold et al. 2004).

To develop a well-known example using this framework, consider a host–parasite system in which two interacting networks exist at the organismal level: the network of hosts and the network of parasites, both of which are closely engaged with each other. In addition to these community- and population-level network interactions, each host and parasite contains within it several more embedded networks of phenotypic, developmental, and genotypic interactions. Evolutionary change in a host characteristic (e.g., an increase in the constitutive investment into an immune response) can alter the connections among and within all the component networks in the system. Host evolutionary change can exert selection on the parasites, which may evolve in response and in turn exert selection on hosts with respect to the phenotypic traits influencing interactions with the parasite. This evolutionary arms race can influence host phenotypic traits that then feed into host–host interaction networks—for example, parasitism affects male nuptial coloration in three-spined sticklebacks, and the resulting variation in nuptial coloration among males provides the substrate for sexual selection exerted by females’ preference for increased male coloration (Milinski and Bakker 1990). Furthermore, parasitic interactions within a host can influence both parasite–parasite and host–parasite interactions, such as when ectoparasites alter host health and lower barriers to secondary bacterial infections (Bandilla et al. 2006; Pedersen and Fenton 2007). In the other direction, changes in host characteristics including parasite tolerance can also affect the abundance of both host and parasite populations (Hassell and Waage 1984; Penczykowski et al. 2016), and the connections among the populations in their community (Wood et al. 2007). Evolutionary changes in host–parasite interactions can also affect other species in the community, both directly and indirectly. For example, increased average parasite load in a predator population decreases host viability and reduces predation pressure on a corresponding prey population, thus affecting that prey population’s interactions with its own food sources.

A key area where this framework has the potential to contribute is with quantifying multilevel selection and the evolutionary ecology of behavior. Current approaches in behavioral ecology have already suggest that behavioral variation is best understood through a multilevel approach, where variation can be partitioned at multiple levels such as the within- and among-individual levels (Dingemanse and Araya-Ajoy 2015; Westneat et al. 2015) in a way that is similar to multilevel selection approach (Goodnight 2005; Eldakar and Wilson 2011). Behaviorists are now increasingly considering the effects of individual–individual interactions on behavior (Dingemanse and Araya-Ajoy 2015) and selection (Formica et al. 2011) at different scales, but less so across different levels of organization. Hierarchically embedded networks can provide a richer characterization of connections across levels, with having more nuanced measures of sublevels (e.g., groups, demes, subpopulations) being particularly important for our understanding of the effects of ecological conditions on multilevel selection regimes. For example, it could allow us to consider the effects of an increase in resource abundance at the level of the landscape on interactions among individuals, groups, demes, and populations as well as the resulting changes in selection (MacColl 2011).

Our examples highlight how changes at a given level of organization can exert influence within and across all levels of organization in the system, and how characterizing connections across levels of organization will help to resolve outstanding questions, such as how multilevel selection operates. To date, most studies remain primarily focused on the effects of change within one such level of organization, or, at most, two adjacent levels. Explicitly thinking about hierarchically embedded networks, where dynamics in a given study system are linked to flow-on changes across the broader environment in which that system is embedded, will encourage a broader and more interdisciplinary general approach to behavioral and community ecology.

## FUTURE WORK

Though the concept of hierarchical embeddedness of networks has been touched upon implicitly or explicitly by a number of subdisciplines in ecology and evolution, accounting for the nested and embedded structure of biological systems in the theory and practice of behavioral ecology and community ecology remains rare. An immediate obstacle is to devise new quantitative approaches to measuring network propagation and indirect connection effects in both theoretical and experimental frameworks. Models could provide testable predictions about how we might expect connections at one scale to influence connections at higher levels, and vice versa (Moore et al. 1997; McGlothlin et al. 2010). While the advantages of interdisciplinary science are clear, one potential challenge is to ensure that all researchers can find a common ground and common goals. This is no trivial task, and the conceptual framework discussed here is in part meant to provide a stepping-stone for quantitative, or even conceptual, integration of nominally different representations of biological interactions.

Finding suitable model systems for studying hierarchically embedded network dynamics is an important future goal, as well. The ideal system should be amenable to experimentation across all levels of organization from genetics, to individuals, to populations and communities. We have highlighted several examples that demonstrate that this is possible. Microbial systems should be especially powerful given the experimental control they provide. Host–parasite systems also offer an obvious link between connections among genes and connections among individuals and across these levels (Milinski and Bakker 1990; Susi et al. 2015; Penczykowski et al. 2016). Finally, the work done on sticklebacks (Box 1) and other teleost fish (e.g., cichlids and guppies) exemplifies how our framework can integrate top–down approaches in natural environments with bottom–up approaches using the genomic tools available for these species.

**BOX 1. A CASE STUDY OF HIERARCHICALLY EMBEDDED NETWORKS OF STICKLEBACKS AND THEIR PREDATORS**

Three-spine stickleback *Gasterosteus aculeatus* are found in aquatic environments across the northern hemisphere. Populations originated in the marine environment and have repeatedly colonized freshwater habitats since the last glacial retreat. The interactions between these populations and a range of predators have shaped numerous aspects of their behavior, morphology, and life histories (Reimchen 1994; McKinnon and Rundle 2002), making them a classic model system in evolutionary biology. We can conceptualize stickleback populations as two broad communities: a marine and a freshwater community. These communities are connected by anadromous stickleback populations that live in marine habitats and migrate to and reproduce in freshwater environments. The two communities differ in their primary predators: marine communities are dominated by large, piscivorous predators, whereas larval odonates (dragonflies and damselflies) are the predominant sticklebacks predators in many freshwater communities (Reimchen 1994).

Stickleback populations among communities differ in several traits, especially those involved in antipredator defense such as behavior and morphology. Conceptualizing the connections among genotypes, phenotypes, populations, and communities as hierarchically embedded networks can offer a fuller understanding of the proximate and ultimate drivers of phenotypic evolution in this system. For example, marine and anadromous sticklebacks are usually more heavily armored than their freshwater counterparts (Bell et al. 1993; Marchinko 2009). From an ultimate point of view, this appears to be driven by differences in predation regimes between the two habitats: heavy plates and spines in marine and anadromous sticklebacks reduce their risk of predation by piscivores, but in freshwater environments where predatory odonates are more common, these plates and spines can actually increase the chances of a stickleback being captured (Bell et al. 1993). Mechanistically, the repeated loss of these morphological defenses is linked to two major genes, Pitx1 and Eda (Colosimo et al. 2005; Barrett et al. 2008; Marchinko 2009). These genes form a gene interaction network within an individual (Peichel 2005), and thus are embedded within phenotypic traits that are contained within the individuals.

A major contribution of hierarchically embedded networks is that this framework can be used to make predictions about how perturbations could cascade through the whole system. For example, overfishing of mesopredators in marine environments is a global problem (Pauly and Palomares 2005). Reductions in marine predator populations could lead to an increase in the adult survival of anadromous sticklebacks in the marine community. This increase could result in more armored individuals returning to the freshwater community, and thus, a higher frequency of alleles that generate defensive morphology. Dragonfly populations are then likely to benefit as they are more successful predators on the more heavily armored sticklebacks, and can have different foraging strategies. If adult dragonflies choose to remain at the location where this armored population is found, this could decrease predation on nearby freshwater stickleback populations. Alternatively, if an increase in the dragonfly population creates a source of dispersing adult dragonflies, this could result in increased predation in other freshwater populations, thus driving stronger selection against alleles encoding defensive spines and plates. In either case, an indirect link can exist between otherwise disparate marine and freshwater stickleback populations, as well as between different freshwater stickleback populations. Further indirect connections exist between the behavior of humans (fishing) and predators (dragonfly foraging decisions). This example demonstrates how exploring hierarchically embedded networks can facilitate discussions among disciplines (in this case conservation specialists, population ecologists, behavioral ecologists, and evolutionary biologists), enable the development of novel hypotheses and testable predictions, and deepen our understanding of proximate and ultimate causes of behavior.

## CONCLUSIONS

A wealth of cutting-edge research informs our understanding of molecular mechanisms, ecological dynamics, and evolutionary processes at different levels of biological organization. We argue that explicitly considering the embeddedness of biological networks will resolve new interdisciplinary questions on how more mechanism-inclined versus ecology and evolution-inclined research programs relate to each other. Among the most pressing obstacles to progress is to bring together multiple fields analyzing the structure of connections at each level of biological organization and from multiple perspectives, which normally operate under different vocabularies and focus on different spatial and temporal scales. We aimed here to develop a step toward tackling this obstacle by providing a conceptual tool that can align different traditions of analysis and enquiry to generate truly novel insights.

## FUNDING

This work was supported by a Deutsche Forschungsgemeinschaft Scientific Network grant (‘The role of interaction structure in eco-evolutionary dynamics (EcoEvoInteract)’, FA 1420/3-1).

We thank Carey Nadell for very thoughtful discussions and suggestions on the manuscript. We also thank two reviewers for their constructive feedback on a previous version of this manuscript.
